# Robotic conformal 4D printing of liquid crystal elastomers

**DOI:** 10.1126/sciadv.aeb2417

**Published:** 2026-02-11

**Authors:** Christopher Chung, Huan Jiang, Alston X. Gracego, Martin L. Dunn, Kai Yu

**Affiliations:** Department of Mechanical Engineering, University of Colorado Denver, Denver, CO 80217, USA.

## Abstract

Four-dimensional (4D) printing of liquid crystal elastomers (LCEs) serves as a promising approach to create reversibly shape-changing structures for diverse applications, including soft robotics, deployable systems, and adaptive surfaces. Central to programming these shape transformations is the spatial alignment of microscale liquid crystal mesogens. Existing 4D printing methods for LCEs are constrained by the alignment of mesogens within 2D planes, thereby limiting the range of achievable deformation. This work presents a robotic direct-ink-writing conformal 4D printing technology that enables deposition of LCEs onto complex, nonplanar 3D substrates. An algorithm is developed to generate printing paths, point normals, and control codes to guide the motion of a six-axis robotic arm. Conformal printing is demonstrated on various surfaces with different printing paths, and the resulting shape-changing behaviors are studied to highlight the unlocked design space. Integration with 3D scanning further allows printing onto substrates with unknown geometries, such as egg surfaces, which enables applications in on-demand protective coatings and structural repair.

## Teaser

A robotic conformal 4D printing method is developed to create LCEs with diverse actuation modes and functions.

## INTRODUCTION

Four-dimensional (4D) printing of shape-changing materials has enabled the fabrication of structures that can actively reconfigure their shapes in response to external stimuli. These programmable and adaptable responses substantially expand the functionality of printed components and drive innovations across a wide range of applications, including morphing structures, soft robotics, biomedical devices, and aerospace systems (*1*–*3*).

Various mechanisms have been explored to realize 4D printing, including viscoelasticity of shape memory polymers (*4*–*6*), swelling of hydrogels (*7*–*9*), mismatched thermal expansion (*10*, *11*), and incorporation of magnetic particles (*12*, *13*). Recently, liquid crystal elastomers (LCEs) have attracted considerable attention because of their large, reversible, and rapid actuation, with specific work capacities comparable to biological muscles (*14*). They also exhibit distinct soft elasticity and excellent energy-absorption capabilities, arising from the rotation of mesogens (two to three linked benzene rings) during deformation (*15*–*18*), which further highlights their potential as promising candidates for protective equipment and biomedical implants.

To date, extrusion-based printing methods such as direct ink writing (DIW) have dominated the 4D printing of LCEs (*19*–*23*). In these approaches, shear forces generated by resin flow within the nozzle align mesogens along the filament axis to enable reversible actuation. More recently, external electric or magnetic fields have been applied during vat polymerization-based 3D printing to control mesogen alignment within printed layers (*24*, *25*). Despite these advances, mesogen alignment in current studies has remained primarily confined to 2D printing planes. Because the spatial distribution of mesogen orientation governs the actuation patterns of LCE structures, existing 4D printed LCE systems have been largely limited to transforming simple planar geometries into 3D forms through basic bending or twisting deformations. Thus, the broader potential of LCEs as transformative actuator materials in complex 4D printing applications remains largely untapped.

In parallel, extrusion-based conformal 3D printing techniques have recently emerged. This printing method enables filament deposition onto nonplanar substrates (*26*–*28*), which overcomes inherent manufacturing constraints of traditional planar printing and offers enhanced design freedom. The development of these techniques often relies on specialized algorithms that generate printing paths in 3D space and advanced motion control systems (e.g., multiaxis robotic arms) that precisely guide filament placement. A common strategy for generating these paths involves projecting desired 2D paths onto conformal 3D surfaces using various mathematical methods (*29*–*31*), such as the Möller-Trumbore intersection algorithm (*32*).

Conformal 3D printing has demonstrated the fabrication of intricate, freestanding structures using fused deposition modeling (FDM) of thermoplastics (*30*, *33*) and DIW of ultraviolet (UV)–curable resins (*34*, *35*). However, its potential to advance LCE-based 4D printing remains largely unexplored. A primary challenge lies in the lack of an effective algorithm that simultaneously generates coordinates and point normal of a desired printing path. Ideally, DIW nozzles for LCE printing should remain perpendicular to the substrate to ensure uniform mesogen alignment and consistent actuation performance. Thus, obtaining point normal is essential for dynamically controlling nozzle orientations during conformal printing. However, current algorithms primarily focus on determining path coordinates alone. Very recently, Armstrong *et al.* (*34*) developed a path-planning algorithm using the Möller-Trumbore approach, wherein both coordinates and normal vectors are calculated at each trajectory point. This method enabled successful DIW deposition of UV-curable filaments on nonplanar substrates in contour-parallel patterns. However, its capability to produce more intricate, arbitrary filament patterns remains unclear, which is crucial for designing complex shape-changing patterns of LCE structures.

This study introduces a robotic conformal 4D printing technology for LCEs that uses a six-axis robotic arm-driven DIW printing system as the manufacturing platform. The development of this technology is supported by an algorithm that simultaneously generates printing paths, point normals, and control codes to guide the motion and rotation of the robotic arm with high precision. It allows user-defined, arbitrary filament patterns within each printing layer that can be precisely controlled during printing. The algorithm is demonstrated by printing LCEs onto complex surfaces explicitly defined by geometric functions, including spherical, saddle, and toroidal shapes, using different filament patterns such as directional-parallel and contour-parallel. The resulting actuation behaviors of the printed LCE structures are characterized and compared with finite element analysis (FEA) predictions. Moreover, the workflow is extended to conformal printing on irregular, undefined surfaces (e.g., the surface of raw eggs) using integrated 3D scanning. The DIW process allows in situ control of the deposition temperature to selectively print LCEs in polydomain or monodomain states, which enables fabrication of LCE structures with programmable actuation, protection, or combined functionalities.

## RESULTS

### Robotic DIW printing of LCEs

**Fig. 1.**
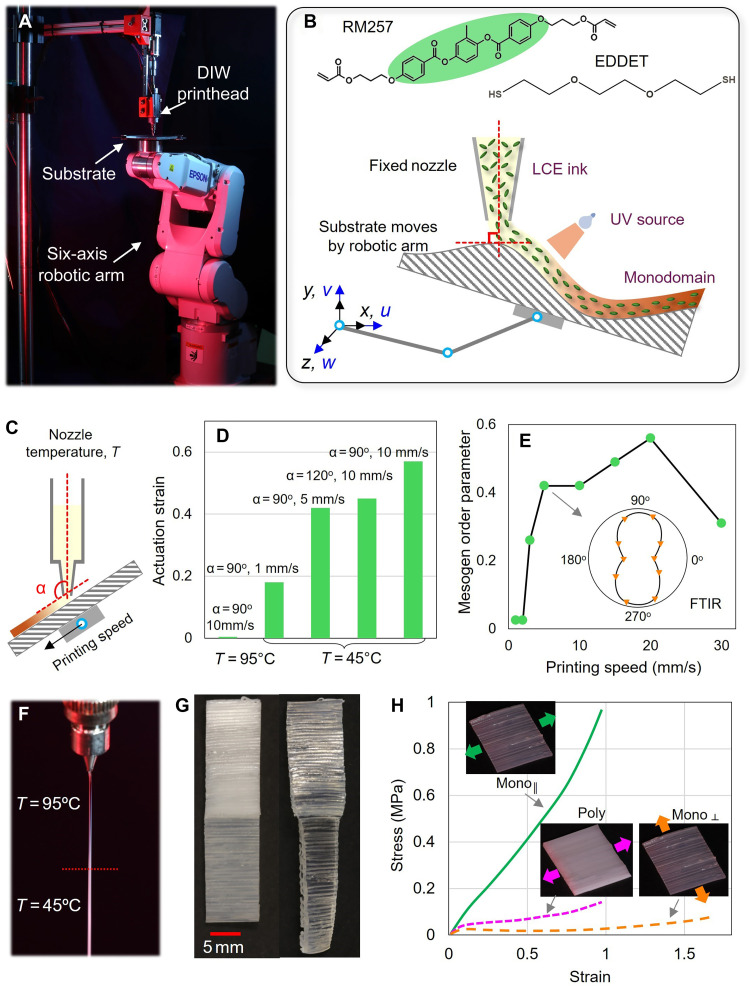
Robotic conformal DIW printing setup and characterizations. (**A**) Printing setup with a fixed DIW printhead while the substrate is attached to the robotic arm. (**B**) Monomers used to synthesize the LCE ink: 4-(3-acryloyloxypropyloxy) benzoic acid 2-methyl-1,4-phenylene ester (RM257) and 2,2′-(ethylenedioxy)diethanethiol (EDDET). During conformal printing, the nozzle is maintained perpendicular to the substrate. HS and SH denote terminal thiol groups. (**C**) Three primary printing parameters that affect the actuation behavior of printed LCEs. (**D**) Summary of actuation strain at 110°C for LCE samples printed with different speeds, nozzle orientations, and temperatures. (**E**) Summary of mesogen order parameter for different printing speeds characterized by polarized Fourier transform infrared (FTIR) spectroscopy. (**F**) Filament extrusion behavior at temperatures below and above the nematic-isotropic transition temperature. (**G**) A printed rectangular sample with polydomain and monodomain halves, where only the monodomain side exhibits shape actuation upon heating. (**H**) Stress-strain relationships of printed monodomain and polydomain LCE samples. Poly, polydomain; Mono_║_, monodomain loaded perpendicular to the mesogen alignment; Mono_║_, monodomain loaded parallel to the mesogen alignment.

Figure 1A shows the printing setup, which integrates a DIW printhead (Hyrel KR2, Norcross, GA, USA) with a six-axis robotic arm (Epson C4, Japan). During printing, the printhead was fixed while the substrate was manipulated by the robotic arm. This arrangement helped avoid continuous movement of a bulky printhead equipped with thermocouple units and a motor for resin extrusion. A thiol-acrylate main-chain LCE system was used for printing (*36*). The printable ink was synthesized from the diacrylate mesogen monomer 4-(3-acryloyloxypropyloxy) benzoic acid 2-methyl-1,4-phenylene ester (RM257) and the dithiol spacer 2,2′-(ethylenedioxy)diethanethiol (EDDET) (Fig. 1B, top). Detailed material compositions and ink preparation procedures are provided in Materials and Methods. The precursor resin was partially cured into oligomers to achieve the desired viscosity before being loaded into the DIW syringe, which features a nozzle with an inner diameter of 0.5 mm. A 405-nm UV laser was used to cure the LCE filaments upon deposition.

During printing, the substrate moving speed is always set equal to the LCE filament extrusion speed to prevent disruption of filament deposition and ensure high-quality printing. Movies S1 and S2 respectively show the printing of LCE samples on flat and oblique substrates. As illustrated in Fig. 1C, the actuation behaviors of printed LCEs are primarily influenced by three parameters: the angle between the nozzle axis and the substrate, the substrate moving speed, and the nozzle temperature. Single-layer LCE samples were first printed onto a planar glass substrate, and their free-standing actuation behaviors were characterized using dynamic mechanical analysis (DMA), with the temperature ramped from −20° to 110°C at 2°C/min. The evolution of actuation strain is shown in the Supplementary Materials (fig. S1, A and B), and the final strain magnitudes at 110°C are summarized in Fig. 1D. Notably, all printed LCE samples returned to their original, as-printed configurations upon cooling, without notable loss in actuation strain over 12 heating-cooling cycles (fig. S2).

First, it was observed that the actuation strain of printed LCEs decreases as the orientation of the DIW nozzle deviates from the perpendicular position relative to the substrate. For example, when the nozzle angle was increased from 90° to 120°, the actuation strain decreased from 58 to 45% at a printing speed of 10 mm/s, likely due to reduced friction at the nozzle tip, which decreases the degree of mesogen alignment. However, when the printing angle was smaller than 90°, the printing quality deteriorated substantially, as shown in the Supplementary Materials (fig. S1C). Although the increased friction at smaller print angles could enhance shear-induced mesogen alignment near the nozzle tip, the unstable printing quality under these conditions led to poor filament deposition and inconsistent sample properties. These observations highlight the importance of maintaining a perpendicular nozzle orientation during conformal printing on curved surfaces to achieve stable filament deposition and consistent actuation performance, as illustrated in Fig. 1B (bottom).

Second, when the nozzle is maintained perpendicular to the substrate, the actuation strain increases with the substrate moving speed, or, equivalently, the filament extrusion speed, due to enhanced shear flow within the nozzle that promotes mesogen alignment. To quantify this effect, polarized Fourier transform infrared (FTIR) spectroscopy was conducted (see fig. S3) to determine the samples’ order parameter (*37*–*39*), which is a scalar between 0 and 1 to characterize the alignment degree of mesogens along the axial direction of the filament. As summarized in Fig. 1E, the order parameter increases from ~0.03 to 0.43 as the printing speed increases from 0.25 to 5 mm/s. Further increases in speed led to only marginal improvements or even slight decreases, likely due to intense acceleration of the substrate and unstable printing conditions. Therefore, unless otherwise noted, a printing speed of 5 mm/s was used in all subsequent experiments.

Last, nozzle temperature plays a notable role in determining the actuation behavior of the printed LCEs. At a temperature of 45°C, which is near the nematic-isotropic transition temperature (*T*_ni_) of the LCE, the printed samples exhibit a monodomain state and repeatable reversible actuation. In contrast, at 95°C, which is well above *T*_ni_, the samples transition into a polydomain state at room temperature without globally aligned mesogens. Visually, the filament changes from transparent to opaque (Fig. 1F). Consequently, the printed samples do not exhibit shape changes upon heating, even at a high printing speed of 10 mm/s (Fig. 1D). Similar trends have been reported by Wang *et al.* (*40*), who have suggested that, at higher printing temperatures, the lower ink viscosity allows the aligned mesogens to rapidly relax back to a random, polydomain state before UV cross-linking. For filaments printed at 45°C, the cross section may also exhibit a monodomain shell and a polydomain core, as reported by Wang *et al.* (*40*). However, due to our relatively small nozzle diameter (0.5 mm), the polydomain core region is expected to be less notable. Therefore, this study focuses on the averaged actuation response of the printed LCE filaments to demonstrate the capabilities of the robotic conformal printing process.

This temperature-dependent behavior offers a unique opportunity to dynamically control material actuation during printing. As demonstrated in Fig. 1G, a single LCE sample was printed with identical printing parameters, except for temperature. The top side was printed at 45°C, after which the printing process was paused. The nozzle temperature was then raised to 95°C, held isothermally for 10 min, and printing resumed to complete the bottom side. Upon heating to 110°C, only the top side (monodomain) shrank, while the bottom side (polydomain) remained unchanged.

Although without reversible actuation, the printed polydomain LCEs exhibit compliant mechanical responses suitable for energy absorption. Figure 1H presents uniaxial tensile test results for LCE samples with identical dimensions but different mesogen alignment states, which are a result of the different printing temperatures (45° or 95°C). When stretched perpendicular to the alignment direction of monodomain LCEs, the samples exhibited soft elasticity characterized by a low stress required to induce strain. This behavior is due to effective mesogen rotation in the direction of external stretch that absorbs energy. Polydomain samples also showed soft elasticity and a slightly higher stress level. In contrast, samples stretched along the alignment direction of monodomain LCEs displayed much stiffer responses without soft elasticity. It should be noted that, while monodomain LCEs exhibit the highest specific energy absorption in the perpendicular direction, they require the external loading direction to be prescribed. While for most impact events with unpredictable loading directions, polydomain LCEs are preferred as they show no directional bias in their mechanical response.

### Path-planning algorithm and overall printing workflow

**Fig. 2.**
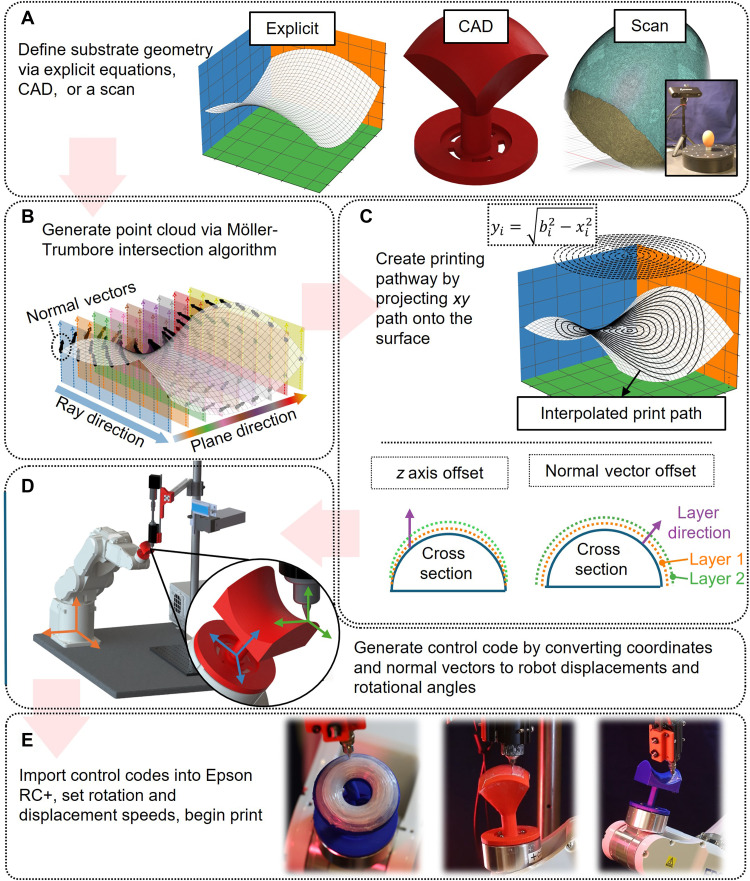
Workflow for executing conformal 4D printing. (**A**) Define the printing surface using a stereolithography (STL) file, generated either from mathematical surface definitions, computer-aided design (CAD) models, or scanning of a physical object. (**B**) Apply the Möller-Trumbore intersection algorithm to generate a point cloud containing both spatial coordinates and corresponding surface normal vectors. (**C**) Project the intended 2D printing paths onto the 3D surface defined by the point cloud. Two methods for multilayer conformal printing are illustrated: the *z*-axis offset method, and the normal vector offset method. (**D**) Determine the spatial coordinates and corresponding Euler angles required to control the robotic arm at each point along the printing path. (**E**) Execute the printing process. The images show snapshots of conformal printing on toroidal, hemispherical, and saddle-shaped surfaces, respectively.

Figure 2 illustrates the overall workflow for generating printing paths and control codes for robotic conformal printing on nonplanar substrates. Compared to existing methods, the key advantage of this algorithm is its ability to lay filaments in arbitrary patterns within each 3D printing layer while simultaneously maintaining real-time control of the relative orientation between the DIW printhead and the substrate surface.

#### Step 1—Defining the substrate geometry

The substrate geometry can be defined through mathematical equations, computer-aided design (CAD) modeling, or 3D scanning of a physical object (Fig. 2A). The geometry is converted into a stereolithography (STL) file, which discretizes the 3D surface into a mesh of triangular elements. The STL file contains the coordinates of the triangle vertices and the normal vectors of each element. In addition, the local coordinate origin of the substrate is recorded as a reference for subsequent steps.

#### Step 2—Generating point cloud on the 3D surface

Evenly spaced sampling points on the substrate surface are identified, including their coordinates and local normal vectors. These data points form a spatial point cloud used for interpolating and generating the desired printing path in the following step. To obtain this point cloud, the Möller-Trumbore algorithm is used to calculate the intersection points between an evenly distributed series of ray vectors and the triangular elements of the STL mesh. As shown in Fig. 2B, when a ray intersects the STL surface, the algorithm returns both the intersection coordinates ($x_{i}^{0}$, $y_{i}^{0}$, $z_{i}^{0}$) and the local surface normal vector ($u_{i}^{0}$, $v_{i}^{0}$, $w_{i}^{0}$) at the point of intersection. The final result is a database containing the spatial coordinates of all points and the corresponding vector field of surface normals. The resolution and accuracy of the point cloud are controlled by the step size of the ray vector increments in the *x* and *y* directions. In this study, both the STL mesh and the corresponding point cloud are generated to represent the substrate surface curvature with a precision of ±0.25 mm.

#### Step 3—Creating the printing pathway

Once the point cloud coordinates and corresponding normal vectors are generated, the printing pathway is defined by the user as a 2D function in the *xy* plane. Various path patterns can be specified, such as zigzag or contour-parallel trajectories, as illustrated in Fig. 2C (top). A curvature minimizing 2D cubic interpolation method is used to project the specified 2D pattern onto the 3D coordinates (*x_i_*, *y_i_*, *z_i_*) and the associated local normal vectors **n**(*u_i_*, *v_i_*, *w_i_*) at discrete nodes along the printing path.

When printing successive layers on a curved substrate, the overall procedure remains the same, but the point cloud representing the substrate surface will be updated layer by layer. Two methods can be adopted (Fig. 2C, bottom). First, the *z*-axis-offset method simply translates each point in the original point cloud vertically along the global *z* axis by a fixed distance equal to the layer thickness, while the associated normal vectors remain unchanged. This approach is computationally efficient and easy to implement, making it well suited for printing samples with relatively small total thickness. However, as more layers are accumulated, the uppermost layers gradually deviate from the original substrate geometry, resulting in a progressively flatter final structure. In contrast, the second normal-vector-offset method updates each point by translating it along its local surface normal direction by the same layer thickness. This approach preserves the overall curvature of the structure and ensures that each layer remains geometrically consistent with the substrate, with the entire shape being scaled outward (or inward) layer by layer along the surface normal direction. The trade-off is the additional computational cost required to recalculate the updated positions using normal vectors at each step.

#### Step 4—Generating control codes for robotic conformal 4D printing

Once the coordinates and surface normal vectors are determined at each point along the printing path, the next step is to generate the control codes for guiding the robotic arm in conformal printing. These control codes require two essential components: the spatial coordinates and the rotational angles of the robotic arm at each point.

Robotic arms generally support multiple modes of operation, including global absolute, tool-relative, and local-relative movements. In this study, the global absolute movement is adopted to control displacement, which allows all movement points to be registered in advance to enhance code-reading efficiency and minimize the accumulation of kinematic errors over time. To use this operation mode, the coordinates obtained in step 3 are adjusted by adding a fixed translation vector (Fig. 2D), which accounts for the offset between the global coordinate origin of the robotic arm and the starting point of the STL model, as specified in step 1. This transformation yields the required spatial coordinates for the robotic arm.

To calculate the rotation angles of the robotic arm, the transformation matrix ***R*** is determined by solving the equation R·k=n, wherein **k** = [0, 0, 1]*^T^* is the initial vector representing the relative orientation between the nozzle and the substrate and **n** is the target orientation vector defined by the local surface normal at each point (as determined in step 4). The transformation matrix ***R*** contains the sine and cosine terms of the Euler rotation angles—α, β, and γ—about the principal axes. These angles are then used to generate the yaw, pitch, and roll commands required for the robotic arm’s motion control.

However, a major pitfall of this approach is that it involves three rotational degrees of freedom, which increases the risk of encountering singularities in the robot’s kinematics. If a singularity occurs, then the printing process must be restarted, resulting in substantial inefficiency. Singularities can also cause dangerously high rotational velocities in individual joints and, in some cases, may even damage the robotic arm. Notably, due to the rotational symmetry of the DIW nozzle about its own axis, rotation around the *z* axis (γ) is physically unnecessary. Therefore, γ is set to zero to reduce the problem to two degrees of freedom to effectively avoid singularities.

However, after this simplification, the original equation R·k=n becomes unsolvable, because it contains three scalar equations but only two unknowns (α and β). To address this, following the method proposed by Armstrong *et al.* (*34*), the problem is reformulated as a minimization task

$$\underset{\alpha ,\beta }{\text{min}}=1-\left[R\left(\alpha ,\beta \right)\cdot k\cdot n\right]$$ (1)

Solving this minimization problem is equivalent to finding a pair of Euler angles that best align the curved print surface to the target vector, which is perpendicular to the fixed nozzle orientation. Last, the Euler angles form the rotational matrix, ***R***, which is used to transform the previously found (*x_i_*, *y_i_*, *z_i_*) to the rotated configuration.

Last, the spatial coordinates and rotational angles computed for each point are uploaded into the Epson RC+ software to control the robotic arm during the printing process. The control code also includes specifications for the printing speeds (5 mm/s) and rotation speeds. Figure 2E shows snapshots of the conformal printing process on toroidal, hemispherical, and saddle-shaped surfaces, respectively.

### Conformal 4D printing on nondevelopable surfaces

With the developed algorithm, conformal 4D printing was performed on nondevelopable surfaces. Unlike developable surfaces such as cylindrical geometries, nondevelopable surfaces allow the fabricated soft LCE samples to maintain their 3D configuration without collapsing after being detached from the substrate.

**Fig. 3.**
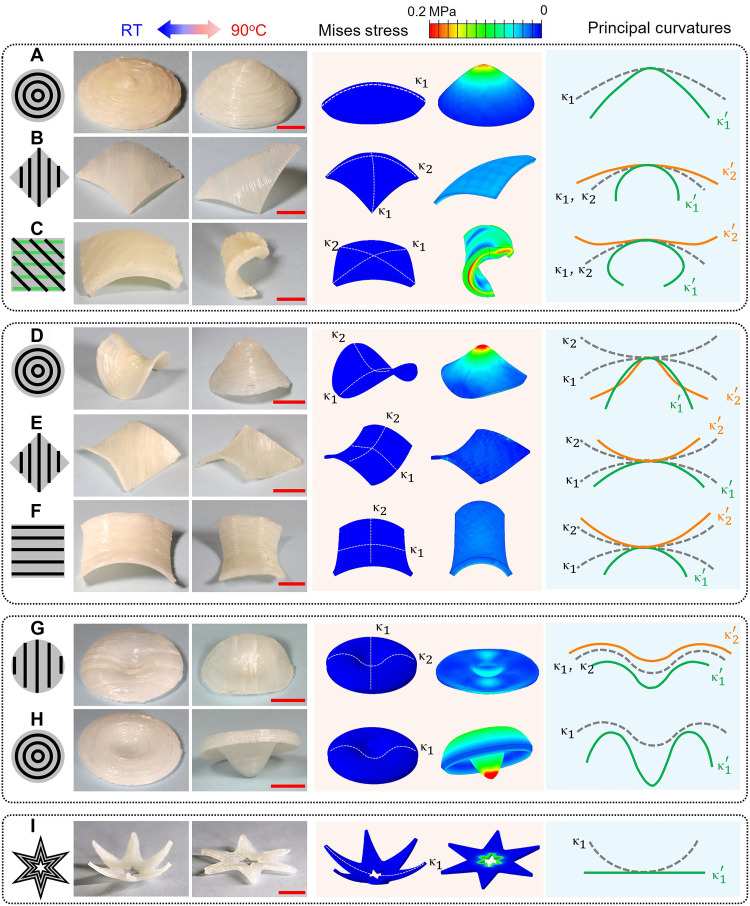
Reversible shape changing of conformally printed LCE samples, including experimental images, FEA analysis, and the deformation of defined paths along the principal curvature direction of the surface. Hemisphere-surface sample with (**A**) circular, contour-parallel printing path, (**B**) filament oriented in the diagonal direction, and (**C**) orthogonal filament directions in the top three and bottom three layers. Saddle-surface samples with (**D**) circular, contour-parallel printing path, (**E**) filament oriented in the horizontal direction, and (**F**) filament oriented in the diagonal direction. Toroidal surface samples with (**G**) filament oriented in one direction and (**H**) circular, contour-parallel printing path. (**I**) A six-pointed star-shaped LCE sample printed on a hemispherical surface with 3D-to-2D shape transition. Scale bars, 1 cm for all figures. RT, room temperature.

As shown in Fig. 3, three representative nondevelopable surfaces were selected: a hemispherical surface with positive Gaussian curvature, a saddle surface with negative Gaussian curvature, and a toroidal surface with zero Gaussian curvature. The substrates were 3D printed using FDM of polylactic acid (PLA) thermoplastic. For each substrate, multilayer LCE samples were conformally printed with two filament laying patterns: directional-parallel (or zigzag) and circular, contour-parallel (see the left schematic in each row). Exemplary printing processes on these three substrates are shown in movies S3 to S7. All printed LCE samples exhibited reversible actuation behaviors when subjected to cyclic heating and cooling between room temperature and 110°C.

To understand how the substrate configuration and filament pattern influence the shape changing behaviors of printed LCEs, FEA was performed in Abaqus, wherein the LCE material was assigned orthotropic thermal expansion. A negative thermal expansion coefficient was used in the monodomain orientation, and positive thermal expansion coefficients were used in the other two directions assuming incompressibility. These values were calibrated from experimental results shown in Fig. 1D. It is also important to note that, instead of using sophisticated constitutive models for polydomain LCEs, the FEA studies treat the LCEs as neo-Hookean solids, with the modulus calibrated from experiments at temperatures above *T*_ni_. This simplification is justified because, at high temperatures, mesogen interactions vanish, and the material essentially behaves like a conventional hyperelastic solid. Detailed descriptions of the modeling process and parameter identification are provided in Materials and Methods and the Supplementary Materials (section S4).

As shown in Fig. 3, the simulation results exhibit good agreement with experimental results in both the magnitude and direction of deformation in the printed LCE structures. Minor deviations are observed, which are likely due to the manufacturing imperfections such as slight deviations in filament placement or the influence of gravitational forces during actuation experiments. However, these differences are negligible relative to the overall sample dimensions. The close agreement confirms that the observed shape changes are primarily driven by the programmed LCE actuation, rather than by any unintended mechanisms introduced during the printing process.

To further elucidate these mechanisms, paths were defined in the model’s initial configuration along the principal curvature directions. Their coordinates were extracted before and after actuation and are compared in the right columns of the figure, where paths in the reference configurations are shown as gray dashed lines, and paths in the deformed configurations are shown as solid orange or green lines.

Figure 3 (A to C) shows LCEs printed on hemispherical substrates. In Fig. 3A, a three-layer sample was printed in a contour-parallel circular pattern. Upon heating, the sample transforms from a dome into a cone driven by in-plane contraction of the LCE filaments along the circumferential direction, which pulls the perimeter inward and pushes the center upward. From FEA, notable stress around 0.20 MPa develops around the apex of the cone. The marked arc path in the initial configuration becomes a parabola-like curve with increased curvature after deformation. In Fig. 3B, a two-layer square-shaped sample was printed in a zigzag pattern with filaments aligned diagonally. Upon heating, contraction along the diagonal causes the sample to lift and fold along that axis, while the perpendicular direction expands due to the Poisson effect. FEA results indicate minimal residual stress after heating, suggesting that the deformation mode induced by the printing direction is compatible with the substrate geometry. The principal curvature along the filament direction increases, whereas it decreases in the transverse direction. In Fig. 3C, the sample consists of six layers with a hybrid filament architecture: The bottom three layers are printed horizontally, and the top three layers are printed diagonally. A top view of the deformed configuration is further provided in the Supplementary Materials (fig. S5A). Upon heating, a competition arises between these printed layers: The top layers tend to fold the sample along the diagonal direction, while the bottom layers favor folding along the horizontal direction. Ultimately, the bottom layers dominate and dictate the primary deformation mode. Slight twisting deformation is also observed with notable residual stress developed at the interface between these two groups of layers. After deformation, one principal curvature increases, while the other decreases and reverses in sign.

Figure 3 (D to F) presents LCEs printed on a saddle-shaped surface characterized by two distinct principal curvatures and directions. In Fig. 3D, the filaments are deposited in a contour-parallel circular pattern. Upon heating, the structure transforms from a saddle into a nonsymmetric dome-like configuration with notable stress around the apex region. The two paths initially defined along the principal curvature directions fold inward after deformation. Figure 3E presents a two-layer sample in which the filaments are aligned along one of the saddle’s principal curvature directions. Upon heating, the overall saddle geometry is largely preserved with minimal residual stress, and both principal curvatures increase. In contrast, when the filaments are placed at an oblique 45° angle relative to the principal curvature directions (Fig. 3F), substantial shear deformation develops, despite both principal curvatures still increasing by similar magnitudes. This effect is clearly illustrated in the Supplementary Materials (fig. S5B), where the sample, initially square in the top view, transforms into a parallelogram after deformation.

Figure 3 (G and H) illustrates LCE samples printed on filled toroidal substrates. In Fig. 3G, the filaments are printed along the vertical direction. Upon heating, the sample undergoes overall vertical contraction and horizontal expansion. The generated stress is moderate (~0.11 MPa), and the two marked paths maintain similar geometry but differ in length. In Fig. 3H, the filaments are printed in a contour-parallel, circular pattern. Upon heating, the structure contracts tangentially along the circular paths, pulling the outer perimeter inward while the central region pushes downward. The final deformed shape resembles an inverted straw hat, with notable stress (~0.19 MPa) developing at the bottom tip.

Overall, the various demonstrations presented in this section highlight the rich design flexibility enabled by conformal printing of LCEs: Samples with identical macroscopic dimensions can exhibit entirely different shape-changing behaviors simply by varying the printing path. The comparisons also reveal key design guidelines: When LCE filaments are aligned with the local principal curvature directions, the printed structures generally preserve similar geometry upon heating (see Fig. 3, A, B, E, and H) with changes only in the magnitude of principal curvatures that determine the final shape. In contrast, when filaments are placed in oblique directions of local principal curvatures, heating induces notable shear deformation in addition to curvature changes (Fig. 3, C and F), which leads to reorientation of the principal curvature directions. In some cases, such as Fig. 3 (D and G), although the filaments are also oriented obliquely, the axisymmetric nature of the geometry allows opposing shear deformations to balance out, and, thus, similar global shapes of the structure are preserved. The developed computational FEA model provides an efficient tool for exploring and designing more complex or application-specific geometries in conformal 4D printing.

Last, conformal printing also enables 3D-to-2D shape transitions of LCE samples. As shown in Fig. 3I, a six-pointed star-shaped LCE sample was printed on a hemispherical surface using two printing layers, with the printing path following contour-parallel trajectories along the boundary of the shape. The central region was intentionally designed to be empty without any deposited filaments. Upon heating to 90°C, the printed structure flattened into a 2D configuration. The deformation agrees well with the FEA predictions, confirming that it results from the contraction of the LCE filaments along the arms of the six-pointed star. The highest stress is concentrated near the central region, where the six arms converge.

### Conformal 3D and 4D printing on undefined surfaces

In many real-world applications, functional polymers are required to be printed onto existing structures for purposes such as structural repair, protective coating, surface reinforcement, and biomedical customization. The target substrates are often intricate with unknown mathematical profiles. To enable conformal printing on such surfaces, the present study uses a 3D scanning approach to capture the as-is surface geometry and generate an STL mesh for path planning.

As an initial demonstration, we printed polydomain LCEs directly onto the surface of a raw egg to create a protective layer. Polydomain LCEs are known for their excellent energy-absorption performance, which stems from the combined dissipation mechanisms of mesogen rotation and network viscoelastic relaxation (*15*). As shown in Fig. 1F, by setting the printing temperature at 95°C, the extruded LCE filaments are cured in the polydomain state. This enables in situ fabrication of a conformal protective coating on a fragile object, providing a streamlined, on-demand approach for safeguarding delicate components.

**Fig. 4.**
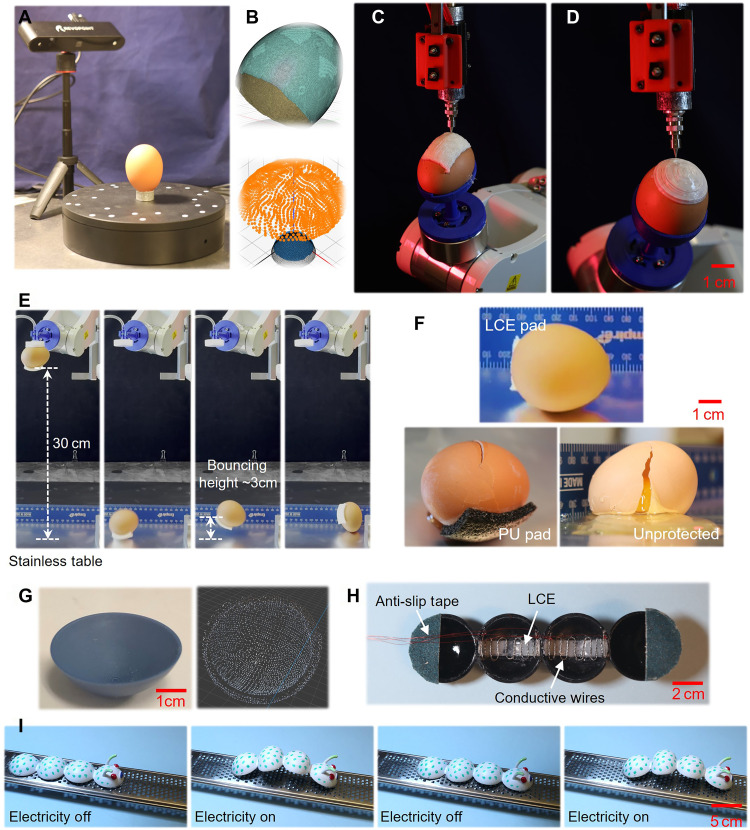
Conformal 3D printing of polydomain LCEs for protection and conformal 4D printing of monodomain LCEs for actuation. (**A**) 3D scanning of a raw egg. (**B**) STL surface mesh and corresponding point cloud. (**C**) Conformal printing of polydomain LCEs on the egg surface using a directional-parallel zigzag pattern. (**D**) Conformal printing using a contour-parallel circular pattern. (**E**) Key snapshots of the dropping and impact process of the LCE-protected egg. (**F**) Postimpact appearance of eggs for three test conditions: protected with an LCE pad, protected with a polyurethane (PU) pad, or unprotected. (**G**) A thermoplastic polyurethane (TPU) hemispherical shell and corresponding point cloud after 3D scanning. (**H**) Interior view of the hemispherical shell showing embedded copper wires. (**I**) Key snapshots of the soft crawling caterpillar during actuation.

As shown in Fig. 4A, a laser-based precision 3D scanner (POP 2 3D Scanner, Revopoint, China) was used to capture the surface of a raw chicken egg. The scanned data was postprocessed in Autodesk Fusion 360 to generate a high-resolution STL file and a point cloud (Fig. 4B). Following the developed path-planning algorithm, six layers of LCEs were conformally printed onto a rectangular region of the egg surface using either a directional-parallel zigzag pattern (Fig. 4C) or a contour-parallel circular pattern (Fig. 4D). The actual printing processes are shown in movies S8 and S9. Due to the precise control enabled by the robotic arm, the LCE filaments were deposited at the intended locations and orientations without damaging the fragile egg.

To evaluate the protective capabilities of the printed polydomain LCE pad, the egg was dropped from a height of 30 cm onto a metal table. Two control cases were tested for comparison: one egg without any protection and another covered with a polyurethane (PU) pad, a widely used commercial protective material. The PU pad has the same thickness as the LCE pad. It is noted that the commercial PU foam was not printed directly onto the egg surface. Instead, it was manually attached and bonded using Super Glue (Loctite Inc., Rocky Hill, CT, USA), which ensured strong adhesion between the foam and the egg. The dropping orientation of the egg was precisely controlled to ensure that impact occurred at the center of the protective pads in all cases.

The dropping and impact process of the LCE-protected egg is shown in movie S10, with key snapshots presented in Fig. 4E. The postimpact appearance of the eggs is shown in Fig. 4F. It was observed that the egg protected by the LCE pad remained completely intact, with no visible cracks or damage following the drop. In contrast, the unprotected egg shattered immediately upon impact (movie S11). The egg covered with the PU foam pad developed an initial crack during the first impact, which propagated and ultimately caused the egg to break during the subsequent rebound and landing (movie S12). Notably, the rebound height of the LCE-protected egg (~3 cm) was notably lower than that of the PU-protected egg, which further demonstrates the superior energy dissipation performance of the polydomain LCE material.

As a second demonstration, we performed conformal 4D printing of monodomain LCEs on the inner surface of spherical shells for soft robotics applications. A 3D caterpillar model was first fabricated using FDM with a soft thermoplastic polyurethane (TPU) elastomer. The model consists of four connected hemispherical shells that form the basic skeleton. In its initial form, the structure resembles a static toy. To impart actuation functionality, monodomain LCE layers were conformally printed along the axial direction inside the middle two hemispherical shells. This process involved scanning the shell geometry using a 3D scanner (Fig. 4G), generating the desired printing path, and performing conformal printing at a nozzle temperature of 45°C. In addition to the LCE layers, copper wires were embedded around each unit to enable electrically induced actuation through Joule heating (Fig. 4H).

This surface coating of a monodomain LCE layer inside the shell introduces reversible shape-morphing capability to the 3D printed model. As shown in Fig. 4I and movie S13, when electrical current is applied, the contraction of the LCE layers causes each hemispherical unit to shrink, resulting in an overall contraction of the caterpillar body. Upon cutting off the electrical input, the system cools naturally via convection, and the body returns to its original shape. By repeatedly turning the current on and off, a crawling motion is achieved on a one-way friction substrate (a stainless-steel grater).

## DISCUSSION

In summary, this work presents a robotic conformal 4D printing technology for LCEs that enables DIW onto arbitrary 3D surfaces with precise control over printing paths and nozzle orientation. These capabilities are realized through the use of a six-axis robotic arm combined with a path-planning algorithm. After studying and optimizing key printing parameters, including nozzle temperature and print speed, conformal printing is demonstrated on a variety of nondevelopable surfaces, such as hemispheres, saddles, and filled toroidal geometries, using different printing paths, including directional- and contour-parallel patterns. The resulting shape-changing behaviors are studied experimentally and further analyzed using FEA to elucidate how printing directions relative to the substrate’s principal curvature directions dictate final shape transformations. The conformal printing process and associated path-planning algorithm are also extended to substrates with unknown profiles by integrating the 3D scanning technique, which enables the deposition of polydomain LCEs for protection and monodomain LCEs for actuation. This capability demonstrates the potential of the approach for on-demand deposition of active polymers in applications such as protective coatings, functional surface modifications, and structural repair.

Although this study focuses on LCEs, the developed conformal printing technology and associated algorithms can be broadly applied to other extrusion-based printing processes and a range of active or structural materials, where precise 3D filament deposition and nozzle orientation control are critical for expanding the structural design space and ensuring high-quality printing outcomes.

In this study, the actuation patterns of conformally printed LCE samples were investigated by selecting representative 3D substrate geometries and printing paths to demonstrate the capabilities of the developed printing process and algorithm. This approach was intended to highlight the effectiveness and versatility of the method. However, the exploration of actuation patterns remains limited in scope. Future work will benefit from adopting systematic design strategies to fully leverage the unlocked design space of conformal 4D printing. For example, integrating topology optimization could allow for the automated discovery of optimized spatial printing paths, leading to LCE structures or composites with unprecedented actuation modes and functionalities.

## MATERIALS AND METHODS

### Ink synthesis for DIW printing

To prepare the printable LCE ink, 20.0 g of RM257, 4.44 g of EDDET, 0.2 g of butylated hydroxytoluene, 0.31 g of 2-hydroxy-4′-(2-hydroxyethoxy)-2-methylpropiophenone, 0.2 g of toluene, and 0.1 g of triethylamine were added to a 100-ml beaker and heated to 80°C for 45 min until all components were fully dissolved. The resulting solution was transferred to a Hyrel KR2 syringe barrel (Norcross, GA, USA) and then left at room temperature to allow oligomerization via the Michael addition click reaction for a minimum of 36 hours before being used in printing. RM257 was purchased from Daken Chemical Limited (Hong Kong, China). All other chemicals were obtained from Sigma-Aldrich (St. Louis, MO, USA) and used without further purification.

### Robotic conformal printing

The DIW printhead consists of a Hyrel KR2 filament extruder, a modified Prusa i3 power supply, and a control board (Prague, Czech Republic). The thermocouple and thermistor of the Hyrel extruder are spliced into the Prusa i3 control board to control the printing temperature and filament extrusion speed via G-code commands. During printing, the printhead was fixed in a vertical position, while an Epson A6 C4 robotic arm (Suwa, Nagano, Japan) was used to manipulate the relative movement of the substrate. A 100-mW, 405-nm UV laser (Mini Dot Diode, Garosa, USA) was integrated with the printhead to cure the LCE filaments after deposition. Substrates used for conformal 3D/4D printing were either standard glass slides or fabricated using a commercial FDM printer (AnkerMake, Changsha, China) with PLA or TPU filaments.

### Material characterizations

The reversible actuation behavior of printed LCE samples was evaluated using a DMA tester. The samples were first stabilized at an initial temperature, followed by a temperature ramp up to 110°C at a rate of 2°C/min. During testing, the samples remained in a free-standing state without an external force applied. Detailed strain evolutions are provided in the Supplementary Materials.

The mesogen order parameter of printed LCEs was characterized using polarized FTIR spectroscopy. Polarized infrared light was transmitted through the LCE sample, and the absorption of the C─H bending mode on the benzene ring was analyzed. The absorption peak is maximized when the polarization direction is parallel to the mesogen alignment and minimized when perpendicular. The degree of mesogen alignment, defined as the order parameter (ranging from 0 to 1), was calculated on the basis of the intensity difference between these two extremes. Detailed results are provided in the Supplementary Materials.

The anisotropic mechanical properties of the LCE lamina were measured via uniaxial tensile tests conducted at room temperature using a universal testing machine (Insight 30, MTS Systems Corp., MN, USA). All tests were performed under a quasistatic condition with a constant loading rate of 10%/min.

### Finite element simulations

FEA was conducted to understand how the orientation of mesogens in 3D space affects the overall shape changing pattern of printed LCEs. Rather than using complex constitutive models, the LCE material was modeled as a transversely isotropic elastomer with distinct thermal expansion coefficients along the axial and transverse directions relative to the mesogen alignment. These coefficients were experimentally determined. For example, the actuation strains of monodomain LCEs were used to determine the axial thermal expansion coefficients, while the transverse coefficients were calculated under the assumption of material incompressibility. Detailed descriptions of the modeling process and parameter identification are provided in the Supplementary Materials (section S4).
