# Supplementary material for: Robotic conformal 4D printing of liquid crystal elastomers
Source: Sci Adv. 2026 Feb 11;12(7):eaeb2417. doi: 10.1126/sciadv.aeb2417 (PMC12893290; doi:10.1126/sciadv.aeb2417)
Supplement: Supplementary file 1 — Supplementary Text Figs. S1 to S5 Table S1 Legends for movies S1 to S13 [file sciadv.aeb2417_sm.pdf]

Science Advances
AAAS

Supplementary Materials for

Robotic conformal 4D printing of liquid crystal elastomers

Christopher Chung *et al.*

Corresponding author: Martin L. Dunn, martin.dunn@ucdenver.edu; Kai Yu, kai.2.yu@ucdenver.edu

Sci. Adv. **12**, eaeb2417 (2026)
DOI: 10.1126/sciadv.aeb2417

The PDF file includes:

Supplementary Text
Figs. S1 to S5
Table S1
Legends for movies S1 to S13

Other Supplementary Material for this manuscript includes the following:

Movies S1 to S13

Supplementary Text

S1. Actuation Strain of Printed Liquid Crystal Elastomers (LCEs)

The actuation strain of printed liquid crystal elastomer (LCE) samples upon heating was characterized using a dynamic mechanical analysis (DMA) tester. Rectangular samples (26 mm in length, 8 mm in width, and 1.5 mm in thickness) were printed along the length direction under different conditions, as shown in **Fig. S1A**. The printing parameters investigated included the nozzle temperature *T*, printing speed *v*, and the relative angle *α* between the nozzle axis and the substrate.

During testing, each LCE sample was first equilibrated at −20 °C for 10 minutes. The temperature then increased to 110 °C at a constant rate of 2 °C/min. The evolution of actuation strain due to shrinkage is presented in **Fig. S1B**. It is observed that all samples begin to develop actuation strain slightly above 30 °C. The final strain magnitude increases with printing speed and is more pronounced when the nozzle remains perpendicular to the substrate. Only the actuation strain during the heating process is reported here for clarity. All samples return to their original length upon cooling, with the expected hysteresis observed in the strain evolution curves.

Monodomain LCEs were successfully printed at a nozzle temperature of 45 °C, which is near the phase transition temperature. At a higher nozzle temperature of 95 °C, the printed LCEs remained in a polydomain state and showed no observable shape change, even at a printing speed of 10 mm/s with a perpendicular nozzle orientation.

Among the tested samples, one was printed on a soft polydimethylsiloxane (PDMS) substrate, while the others were printed on standard glass slides. Printing on the softer PDMS substrate resulted in a reduced actuation strain. This reduction is likely due to the lower substrate stiffness, which results in mesogen relaxation after extrusion and decreases the degree of alignment. This observation is important for accurately simulating multilayer LCE shape changes using FEA, as will be described in the following section.

In general, printing with an oblique nozzle orientation reduces actuation strain under equivalent conditions. In addition, when α is smaller than 90°, the printing quality deteriorates significantly. In **Fig. S1C**, even a small deviation of the nozzle orientation from 90° to 80° leads to poor printing quality with misaligned filament deposition and visible inter-filament gaps.

S2 Actuation of Printed LCE under Repeated Heating–Cooling Cycles

To examine the stability of actuation performance, LCE samples printed at a speed of 5 mm/s were subjected to 12 heating–cooling cycles. The temperature was varied between room temperature and 100 °C at a rate of 2 °C/min. **Fig. S2** shows the evolution of actuation strain and temperature over these cycles. The results indicate that the LCE exhibits highly consistent actuation strain throughout the extended testing, confirming its reliable and stable actuation performance.

S3 Polarized Fourier Transform Infrared Spectroscopy

Polarized Fourier-Transform Infrared Spectroscopy (FTIR) was conducted using a Nicolet iS50 FTIR spectrometer (Thermo Fisher Scientific, Waltham, MA, USA) to measure the mesogen order parameter of LCE samples printed at different speeds. The sample thickness was maintained below 0.5 mm.

During measurement, the LCE samples were kept fixed, and infrared light passed through the sample. The FTIR instrument was equipped with a built-in zinc selenide (ZnSe) polarizer, which can rotate the light polarization direction in 1° increments up to 180°. The stretching of C–H bonds

on the mesogen was used as the signature bond. The absorption peak was highest when the polarization was perpendicular to the mesogen alignment and lowest when it was parallel.

Fig. S3 shows the area of the C–H bending absorption peaks at different polarization angles. At low printing speeds, the plot is nearly circular, suggesting almost identical absorption in different directions and negligible mesogen alignment in the printed LCE. As the printing speed increases, the plot gradually transforms into a dumbbell-like shape, indicating stronger mesogen alignment along the long-axis direction.

The differences in the absorption peaks are used to identify the mesogen order parameter, S. Specifically, the maximum and minimum absorption peaks of the C-H bonds, A_{max} and A_{min}, are used to calculate the dichroic ratio of the anisotropic LCE network as $D = A_{max}/A_{min}$. The order parameter, S, is determined as $S = (D-1)/(D+2)$. The data of the mesogen order parameter is reported in **Fig. 1E** of the main manuscript.

S4. Finite Element Simulations

Finite element analysis (FEA) simulations were conducted in this study to reveal the stress distribution and deformation of principal curvature paths within printed LCEs during shape transformation. Instead of employing sophisticated constitutive models of polydomain LCEs, the FEA study treated the LCEs as elastomeric solids with transversely isotropic thermal expansion coefficients. These coefficients were directly implemented in ABAQUS through user-defined orthotropic expansion coefficients (α_1, α_2, α_3) in principal material directions, where α_1 corresponds to the printing direction, and $\alpha_2 = \alpha_3$ correspond to the transverse directions.

The thermal expansion coefficients were calibrated based on experimental data. For example, as shown in **Fig. 1D**, when the printing speed was 5 mm/s with a perpendicular deposition nozzle, the LCE actuation strain increased by $\varepsilon_1 = -39\%$ from room temperature (25 °C) to 110 °C. This corresponds to a negative, linear expansion coefficient of $\alpha_1 = -0.46\%/°C$. The two transverse expansion coefficients were assumed equal and calculated based on the incompressibility condition of the material. Specifically, the transverse strain was given by $\varepsilon_2 = \varepsilon_3 = 1/\sqrt{1+\varepsilon_1} - 1 = 28\%$, which yield expansion coefficients of $\alpha_2 = \alpha_3 = 0.33\%/°C$.

During FEA, coupled temperature-displacement analyses were performed, and the model was meshed using brick elements. **Fig. S4A** shows the reversible shape changing of a two-layer LCE sample printed on a spherical surface, with filaments oriented diagonally. **Fig. S4B** shows the FEA model after assigning orthotropic expansion coefficients. Initially, identical expansion coefficients were assigned to both LCE layers. However, the simulation results significantly deviated from the experimental observations (**Fig. S4C**). It was later determined that the discrepancy arose because the top LCE layer was printed onto the bottom soft LCE layer, whereas the bottom layer was printed onto rigid plastic. As a result, the two layers exhibited different mesogen alignments and actuation strains. As shown in **Fig. S1B**, when LCE was printed on a soft PDMS substrate, the actuation strain from room temperature to 110 °C increased by only 30%. Accordingly, the expansion coefficients for the top layer were determined to be $\alpha_1 = -0.35\%/°C$ and $\alpha_2 = \alpha_3 = 0.23\%/°C$. These parameters are summarized in Table S1. With these adjusted parameters, the model produced predictions closely matching experimental results (**Fig. S4D**), in terms of both the magnitude and direction of deformation in printed LCE structures.

S5. Top Views of LCE Samples Shown in Figs. 3c and 3e

To better visualize the 3D deformation of the LCE samples shown in **Figs. 3C** and **3E**, additional top views were obtained from the FEA simulations.

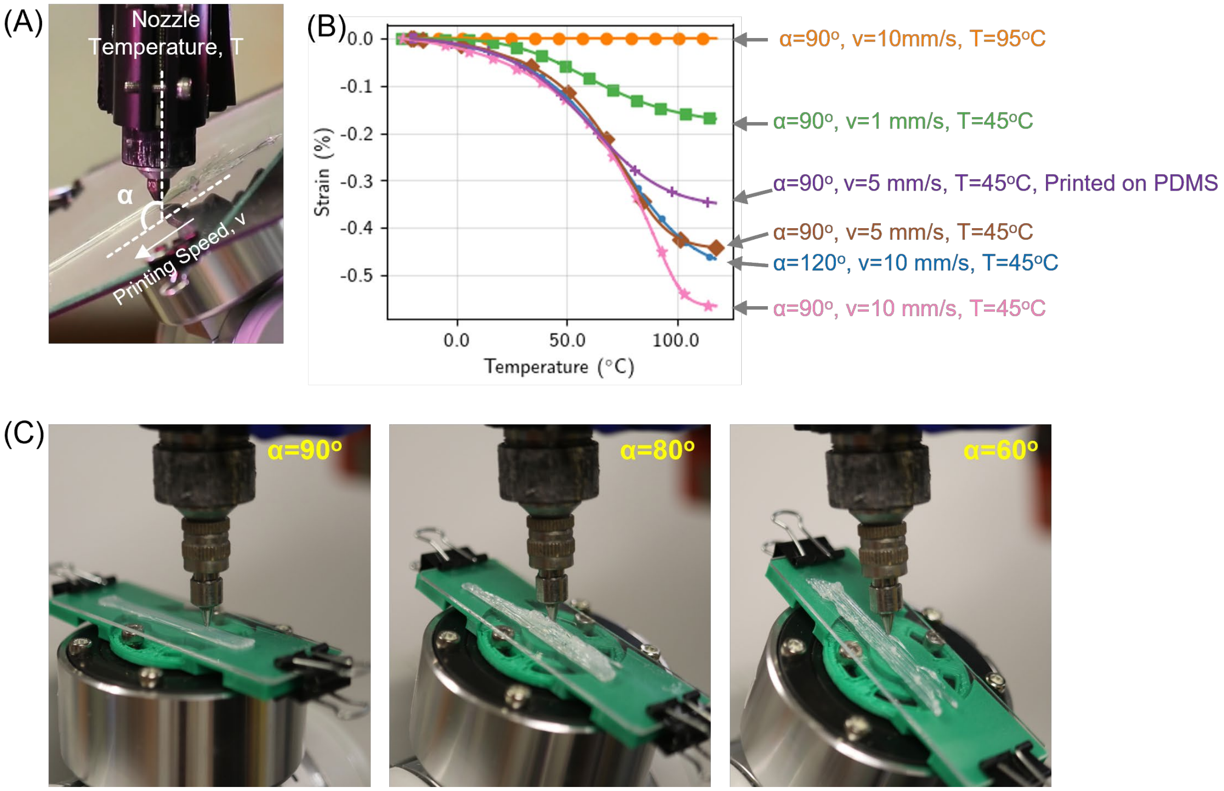

Fig. S1. **Direct ink writing (DIW) printing and actuation strain of liquid crystal elastomers (LCEs).** (**A**) Printing of LCEs on a glass slide, with the three printing parameters marked in the figure. (**B**) Evolution of actuation strain during heating for samples printed under different conditions. (**C**) Appearance of printed LCE filaments when using an oblique nozzle angle.

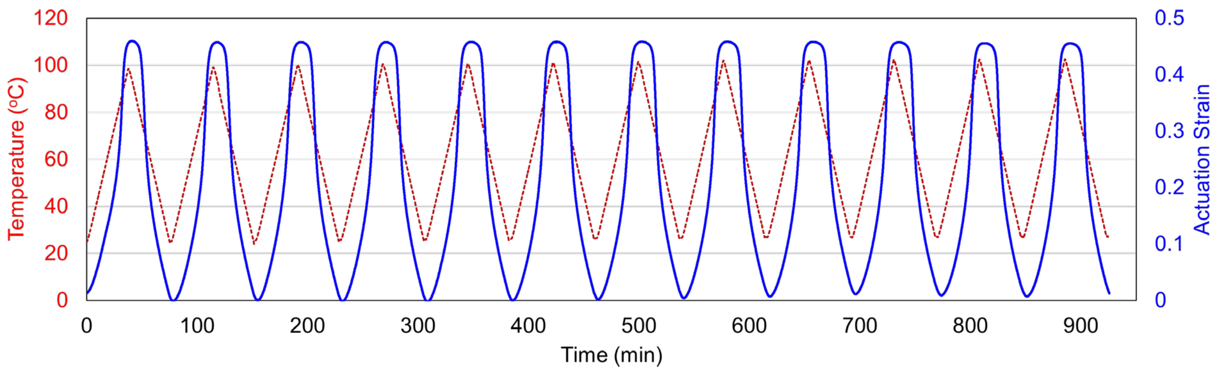

Fig. S2. Evolutions of actuation strain and temperature during the repeated heating-cooling cycles.

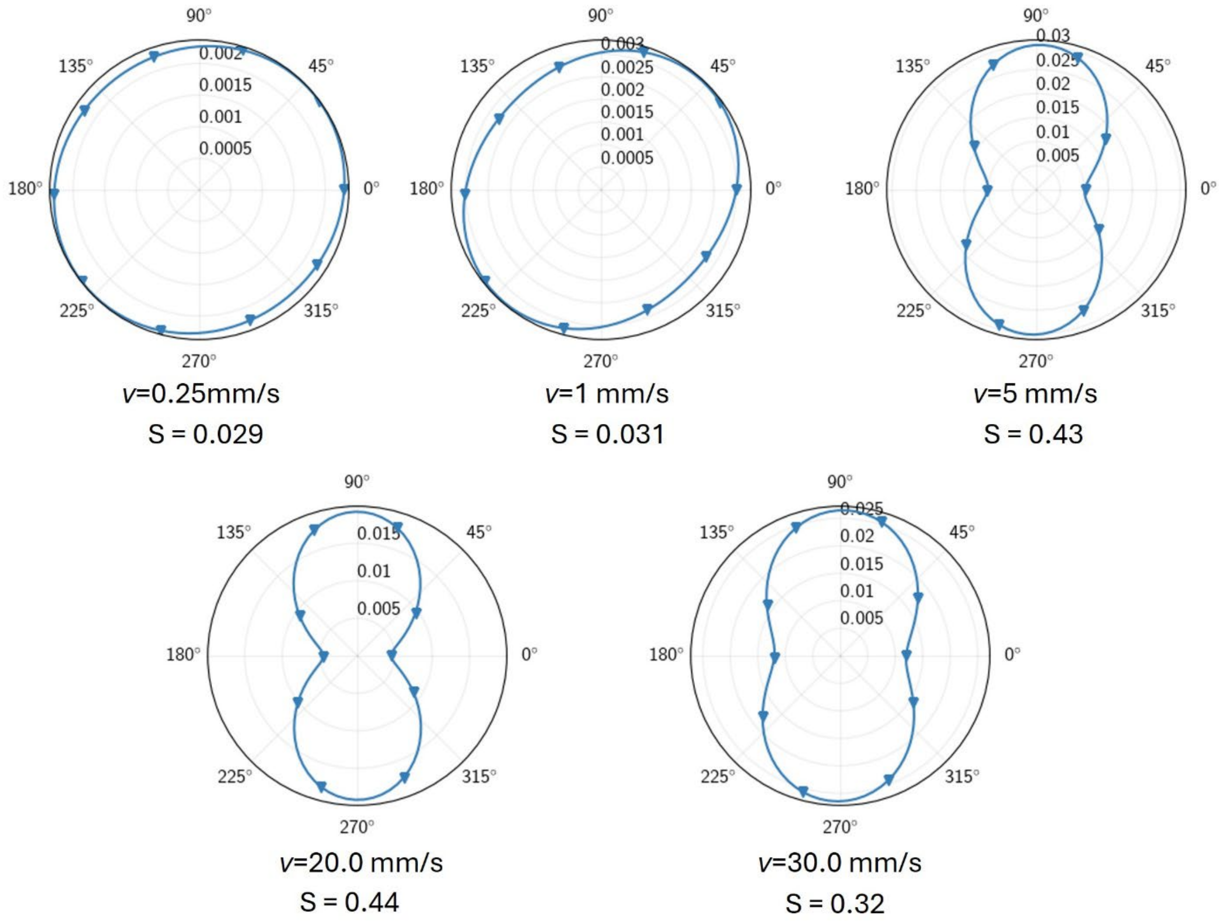

Fig. S3. Polar plots of C–H stretching absorption intensity as a function of light polarization angle for different LCE samples printed at different speeds.

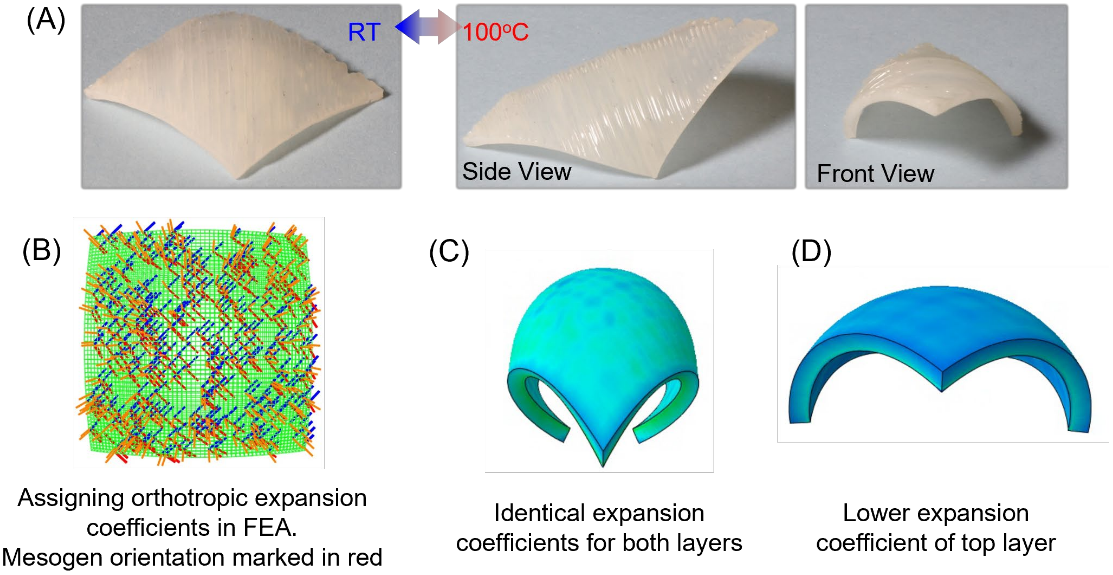

Fig. S4. Reversible shape changing of a two-layer liquid crystal elastomer (LCE) sample printed on a spherical surface. (**A**) Experimental pictures. (**B**) Finite element analysis (FEA) model after assigning orthotropic expansion coefficients. (**C**) FEA predictions with identical expansion coefficients for both layers. (**D**) FEA predictions with calibrated expansion coefficients for the top layer.

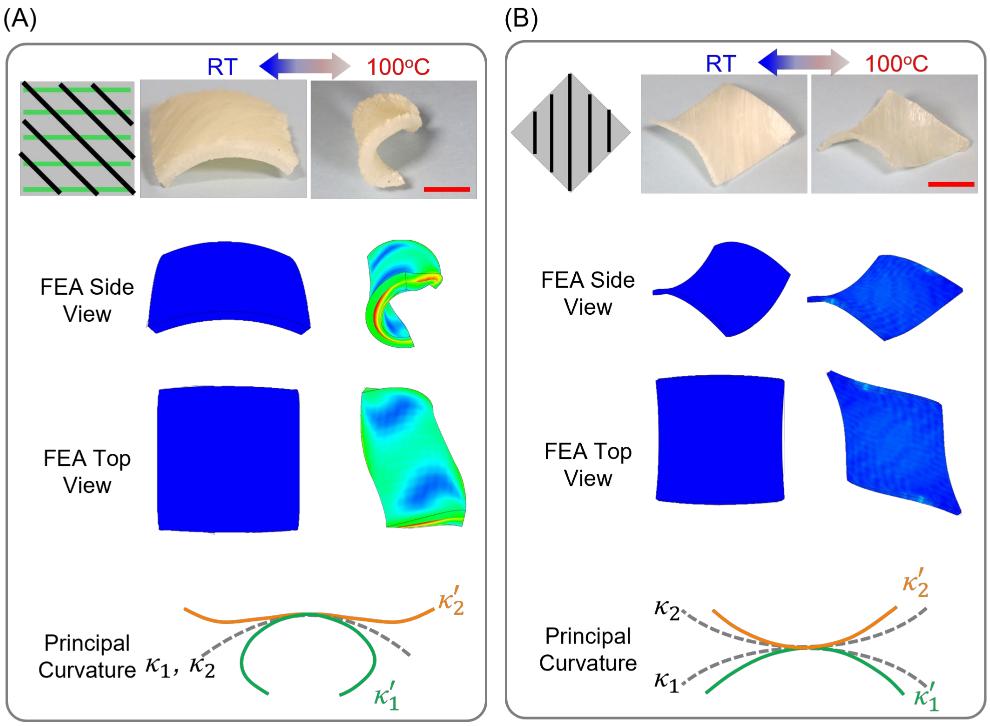

Fig. S5. Experimental images, finite element analysis (FEA) side views, and top views of liquid crystal elastomer (LCE) samples. (**A**) LCE sample printed on a hemispherical surface. (**B**) LCE sample printed on a saddle-shaped surface.

	**α_1 in the printing direction**	**$\alpha_2 = \alpha_3$ in the transverse direction**
**First layer printed on the rigid substrate**	–0.46%/°C	0.33%/°C
**Subsequent layers printed on soft LCEs**	–0.35%/°C	0.23%/°C

Table S1. Orthotropic expansion coefficients used for finite element analysis (FEA) of multi-layer liquid crystal elastomers (LCEs).

Movie Captions

Movie S1. Printing LCE samples on a flat substrate
Movie S2. Printing LCE samples on an oblique substrate
Movie S3. Printing LCE samples on a hemispherical surface in a zigzag pattern
Movie S4. Printing LCE samples on a saddle surface in a contour-parallel pattern
Movie S5. Printing LCE samples on a saddle surface in a zigzag pattern
Movie S6. Printing LCE samples on a toroidal surface in a zigzag pattern
Movie S7. Printing LCE samples on a toroidal surface in a contour-parallel pattern
Movie S8. Printing LCE samples on the surface of a raw egg in a zigzag pattern
Movie S9. Printing LCE samples on the surface of a raw egg in a contour-parallel pattern
Movie S10. Dropping tests of a raw egg protected with an LCE surface layer
Movie S11. Dropping tests of a unprotected raw egg
Movie S12. Dropping tests of a raw egg protected with PU foam
Movie S13. Crawling motion of the caterpillar-like soft robot
